# Improving the introduction of telemedicine in pre-hospital emergency medicine: understanding users and how acceptability, usability and effectiveness influence this process

**DOI:** 10.1186/s12873-024-01034-6

**Published:** 2024-07-12

**Authors:** Seán O’Sullivan, Jennifer Krautwald, Henning Schneider

**Affiliations:** Faculty of Health Sciences, Technische Hochschule Mittelhessen, Gießen, Germany

**Keywords:** Telemedicine, Telehealth, Emergency medicine, Emergency medicals services, EMS, Prehospital, Usability, Human factors, eHealth, Mobile application

## Abstract

**Introduction:**

Increasing numbers of ambulance calls, vacant positions and growing workloads in Emergency Medicine (EM) are increasing the pressure to find adequate solutions. With telemedicine providing health-care services by bridging large distances, connecting remote providers and even patients while using modern communication technologies, such a technology seems beneficial. As the process of developing an optimal solution is challenging, a need to quantify involved processes could improve implementation. Existing models are based on qualitative studies although standardised questionnaires for factors such as Usability, Acceptability and Effectiveness exist.

**Methods:**

A survey was provided to participants within a German county. It was based on telemedical surveys, the System Usabilty Scale (SUS) and earlier works describing Usability, Acceptability and Effectiveness. Meanwhile a telemedical system was introduced in the investigated county. A comparison between user-groups aswell as an exploratory factor analysis (EFA) was performed.

**Results:**

Of *n* = 91 included participants *n* = 73 (80,2%) were qualified as emergency medical staff (including paramedics *n* = 36 (39,56%), EMTs *n* = 28 (30,77%), call handlers *n* = 9 (9,89%)) and *n* = 18 (19,8%) as emergency physicians. Most participants approved that telemedicine positively impacts EM and improved treatment options with an overall Usabilty Score of 68,68. EFA provided a 3-factor solution involving Usability, Acceptability and Effectiveness.

**Discussion:**

With our results being comparable to earlier studies but telemedicine only having being sparsely introduced, a positive attitude could still be attested. While our model describes 51,28% of the underlying factors, more research is needed to identify further influences. We showed that Usability is correlated with Acceptability (strong effect), Usability and Effectiveness with a medium effect, likewise Acceptability and Effectiveness. Therefore available systems need to improve. Our approach can be a guide for decision makers and developers, that a focus during implementation must be on improving usability and on a valid data driven implementation process.

**Supplementary Information:**

The online version contains supplementary material available at 10.1186/s12873-024-01034-6.

## Introduction

With numbers of emergency calls increasing for Emergency Medical Services (EMS), overcrowding being an issue in Emergency Departments (ED) and generally observing an increasing workload in Emergency Medicine (EM), new solutions and approaches are needed to meet these challenges. Current strategies include increasing the number of staff and resources, but with rising costs for the general medical system [1–7]. Therefore new technologies and structures need to be evaluated and introduced to solve these challenges and prevent a loss of performance in EM.

Technologies such as telemedicine are increasingly being introduced and implemented into regular care and treatment processes. As such a technology can deliver health-care services by bridging large distances and connecting remote healthcare providers with each other and/or patients by using modern communication technologies [8].

### Germany EMS and telemedicine

In German EM a nationally available solution hasn’t been established yet while existing systems only provide support in regional or rural areas [7, 9, 10].

These systems often only provide the option of medical support in the pre-hospital field and do not connect to EDs or other specialist structures like a heart catheter laboratory. Although technically this is possible, could improve treatment options and response times [11–13].

With Germany being a physician-based EM System, the mainly available telemedical systems only provide solutions in which an EM physician is not rapidly available but required by paramedics for the treatment of the patient’s condition. Therefore a telemedical EM physician (TEP) can be requested to support paramedics. Of the initial research projects some have now been adapted to regular practice and can even provide long-term data [9, 14–16], but still only represent certain regions in Germany.

As the possibilities and capabilities especially in the field of pre-hospital EM are increasingly being understood, especially for time-critical scenarios, the area of non-time critical emergencies (so called non-emergencies) seems to be rather understudied [17–19].

### Telemedical networks

Current German law and medical legislation require that patients in the pre-hospital field need to be seen by a physician, ideally a GP, while these are also faced with increasing amounts of patients and structural changes [20, 21]. A recent survey in which not only physicians but also local german politicians and administrators where questioned, showed that these seemed to support the idea of using telemedicine and limit its application not only to rural areas [16, 22].

Therefore a telemedical solution could not only provide a replacement for an in-person visit, but should be developed as a digital network to connect patients with adequate healthcare providers. This could allow the treatment of critical and non-critical patients within a network.

Concepts like the Emergency Talk Network (ETN) even go further and involve specialist structures like paediatricians in a digital emergency medical network [22]. While ECGs can be transferred using various telemetric devices and healthcare networks seem to improve the treatment f.ex. of acute coronary syndromes, even the leading cardiovascular societies recommend the development and availability of telemedical systems and approaches. [12, 13]

Extending this approach to involve not only one but many more specialists in one network could therefore optimise the flow of patients, the use of available limited resources, improve patient safety as well as guideline adherence even more.

### Users of telemedical systems in Germany

In a nationwide german survey paramedics and emergency physicians approved of the integration of modern technologies to improve processes and treatments [10]. Also in an even older survey from 2012 - during the time of a broad implementation of telemedicine in EM structures in Aachen, Germany - paramedics described that telemedicine is not only seen as a tool to control but rather to supervise and improve therapies. Especially for critically ill or when specialist expertise was needed. Generally processes as well as communication between paramedics, dispatch centres and hospitals seemed to improve [23].

With this technology having been attributed positively for a long time, this must have been achieved by developing systems that would receive high “Acceptability” ratings while also being reliable. Therefore understanding the basis on why there has been such a positive attitude has neither been researched nor quantified with standardised methods. Gaining a better understanding could improve implementation processes and advance the integration of more EM providers and specialist, as there still seems to be factors limiting the integration of telemedicine in many healthcare systems.

### Implementation, continuity and understanding users

But as reported for the field of pediatric EM not only technical aspects like feasibility or reliability but also a lack of knowledge seems to be a challenge [24, 25].

Therefore healthcare regulators should not only support the development of technical connections, interfaces or provide initial financial investments, but continuously invest in continuous training programs of involved staff.

Users of such telemedical systems are mainly paramedics and physicians. Both groups are faced with the challenges of the introduction of a new technology in an already stressful work environment with constantly changing surroundings.

This also extends to aspects likeuser demographics, suitable use-cases but also the individual healthcare providers needs [26, 27].

To understand these Sauers-Ford et al. investigated these using qualitative methods for the application of telemedicine in a paediatric ED: In a connected model the authors described that acceptability of a telemedical system influenced its perceived usability, while usability influences its effectiveness. But also its perceived usability and effectiveness feedback and influence the perceived acceptability.

These aspects “Usability”, “Effectivity” and “Acceptance” which are defined and originate from the fields of user-centered development and computer technologies have only been described in a qualitative approach in EM, although a quantitative approach is regularly used in software development [28–30].

As this has not been described yet (to our knowledge) for the field of pre-hospital EM, we planned on investigating this as such. an approach could improve implementation and integration of telemedicine in EM.

Therefore we saw the need to gain insights on understand the underlying challenges by investigating these in a German county that was currently in the process of introducing a new telemedical system.

## Methods

### Test region

The German county Main-Taunus Kreis (MTK) is a sub-urban region with a mixed population density in the federal state of Hesse. With a very densely city-like population in one half of the county and the other being more rural [31], it provides a challenging region to introduce new emergency medical structures.

Located near the city of Frankfurt am Main with an availability of multiple trauma centres, two university hospitals and many academic teaching centers patients, can rapidly be transported and treated at highly specialised facilities [31].

### New telemedical system

Paramedics at the site of emergency can request support from a TEP. The TEP can access vital signs which are provided via the combined monitoring and defibrillator system Corpuls C3 from the company Corpuls. With the provided software application corpuls.mission paramedics can optionally consult a TEP using live audio and visual communication [32, 33].

As the process of implementation was planned as a step-wise process, not all ambulances were directly equipped. Only ambulances from two of five possible ambulance stations were equipped with the system during the time of the trial. Overall these were 4 of 11 ambulances.

**Fig. 1:**
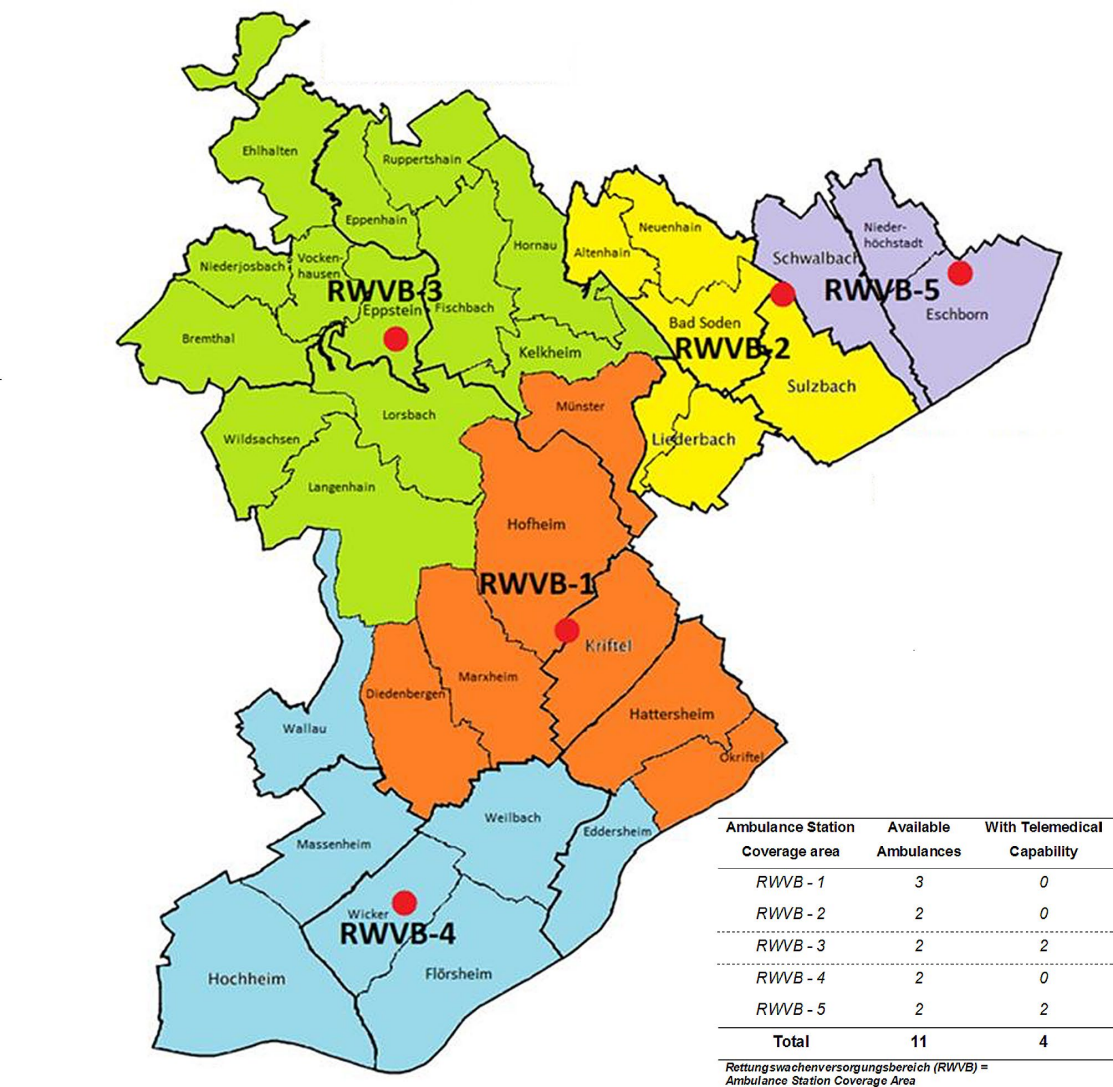
Ambulance stations and ambulances in the county Main-Taunus Kreis during the time of study - original image “Abbildung 25: Rettungswachenversorgungsbereich MTK” page 119 [31] modified by the author by adding the table

2 were located in a rural area, while the other 2 were located in a densely populated area as can be seen in the following Fig. 1.

### Trial design

To understand the involved processes as well as the users’ opinions a pre-test was performed for one week in March followed by a revision and update of the questionnaire. A final questionnaire was then available via an Online-Link from April 3rd 2023 to May 14th 2023. Participants received an invitation via email from the county’s medical director of prehospital EM (German: Ärztlicher Leiter Rettungsdienst = ÄLRD) and could voluntarily participate. During this time the medical director of prehospital EM sent two reminders to the participants to complete the survey.

Results could only be included if participants worked within this county in the field of EM, the questionnaire was fully completed and a data-protection waiver according to EU-GDPR was approved by the participant. Other results were excluded.

### Pre-test

Before the final questionnaire was launched, a pretest was performed for one week in march 2023. Participants were invited to answer the first version of the questionnaire and provide comments.

Participants of this pre-test were medical directors of prehospital EM from several counties of the state of Hesse, the hessian ministry of social affairs responsible for the field of EM and telemedicine (HMSI), paramedics, EMTs, emergency physicians as well as qualified call handlers for emergency dispatch centres.

The participants had to work in regions in which telemedicine in EM was available. These were defined as the counties Giessen and Main-Kinzig-Kreis in Hesse. Other results were excluded. Cronbachs Alpha was analysed with an α-value of 0,05 [34]. After reviewing the results and comments the authors decided if parts of the questionnaire had to be adapted. Only if both authors SO and JK agreed, a change could be performed. If only one agreed, the other author HS would be involved and a change was performed if the majority approved. Between the pre-test and the finale questionnaire a time of 2–3 weeks was planned for revision of the final questionnaire.

### Questionnaire

The final questionnaire consisted of 50 items in german language with single and multiple choice questions as well as Likert scaled and open-ended answer possibilities, which can be viewed in **Annex 1**. These were divided in to 4 parts:

Part A - General Part - consisted of 5 questions regarding age, identified sex, field of work, current qualification and work experience.

Part B - Tele Emergency Physician Concept - consisted of 29 questions regarding the use of telemedicine in emergency medicine with a 5-point Likert scale.

14 of these items were based on the questionnaire from Metelmann et al., 3. From Kuntosch et al. [35, 36] and 11 were adapted after reviewing the results of the pre-test.

Part C - Usability - were 10 questions of the System Usabilty Scale (SUS) in German [37, 38].

Part D - Open Questions - were 6 Open-Ended Questions of which 4 were based on Sauers-ford et al. [28] and 2 on the results and comments of the pre-test.

### Participants

Participants of the final questionnaire had to be medically qualified to work in the field of EM, meaning that they had to be Paramedics, EMTs, Emergency Physicians or be qualified call handlers for emergency dispatch centres. Otherwise participants were excluded.

### Data analysis

Before analysis all data was transferred from the online questionnaire platform to a database (Microsoft Excel, Version 22.10, Vermont, USA [39]). RStudio (2023.12.1 + 402 including R version 4.3.2 (2023-10-31)) was used for statistical analysis as a combined quantitative and qualitative process was planned:

### Quantitative approach

The groups were to be compared depending on qualification, sex, availability of a telemedical system and level of knowledge about telemedicine.

For Analysis of the SUS results the t-test and for more than two groups an ANOVA Test was performed. Significant p values were defined at *0.05*.

T-test was performed for the groups sex, physician vs. non-physician and the availability of a telemedical system. ANOVA was performed for the other groups regarding qualifications, age groups and experience.

An analysis for homogeneity of Variance was performed using Leven’s test (*p* < .05). If Variance of homogeneity was not proven a Welch Test was performed [40].

For significant results a further analysis was performed with the Bernoulli Post-Hoc Test (*α-*value *0.05*).

For correlation- analysis, Pearson’s product-moment correlation was used and *α-*value was defined at *0.05.*

### Factor analysis

The qualitative analysis was performed using an exploratory factor analysis (EFA). With regards to the earlier described qualitative results from Sauers-Ford et al. the factors “Acceptability”, “Usability” and “Effectiveness” [28] were identified and analysed [41–43]:

Further methodical information on the EFA can be viewed in Supplement 1.

Following the results of the Factor Analysis a multiple regression analysis was performed. Based on the results from Sauers-Ford et al and an earlier performed correlation analysis “Acceptably” would be analysed as a dependent and “Usability” and “Effectiveness” as independent variables.

### Multiple regression analysis

The multiple regression analysis included the Mann-Whitney-U- and Kruskal-Wallis-Test as non-parametrical methods.

For significant results of the Kruskal-Wallis-Test, a Bonferroni-Post-Hoc test was performed. The *α-*value was defined at *0.05.* To ensure normal distribution a Kolmogorov-Smirnov-test was performed beforehand with a *p-*value defined at *0.05*. If a significant group difference was to be found the effect size would be analysed using Pearson’s correlation coefficient (*r*) [44].

All methods were carried out in accordance with relevant guidelines and regulations.

As no potential harm was to be expected, the local ethics committee (University Hospital Giessen, Germany) solely required informed consent including a data privacy agreement from the participants.

## Results

### Pre-test

Overall *n* = 19 participants (8 Paramedics, 6 Emergency Physicians, 3 EMTs and 2 call handlers) took part in the Pre-Test. Reliability was confirmed with Cronbachs Alpha and an α-value of 0,837.

The average SUS Score was 74,2% and was also confirmed with an α-value of 0,898. This showed a satisfying internal consistency.

### Changes

As some questions used a past tense adaptations had to be made f.ex in to the present tense. The adaptation can be seen in **Annex 1**, which also includes an explanation for each changes.

### Final questionnaire

#### Participants and qualifications

**Fig. 2:**
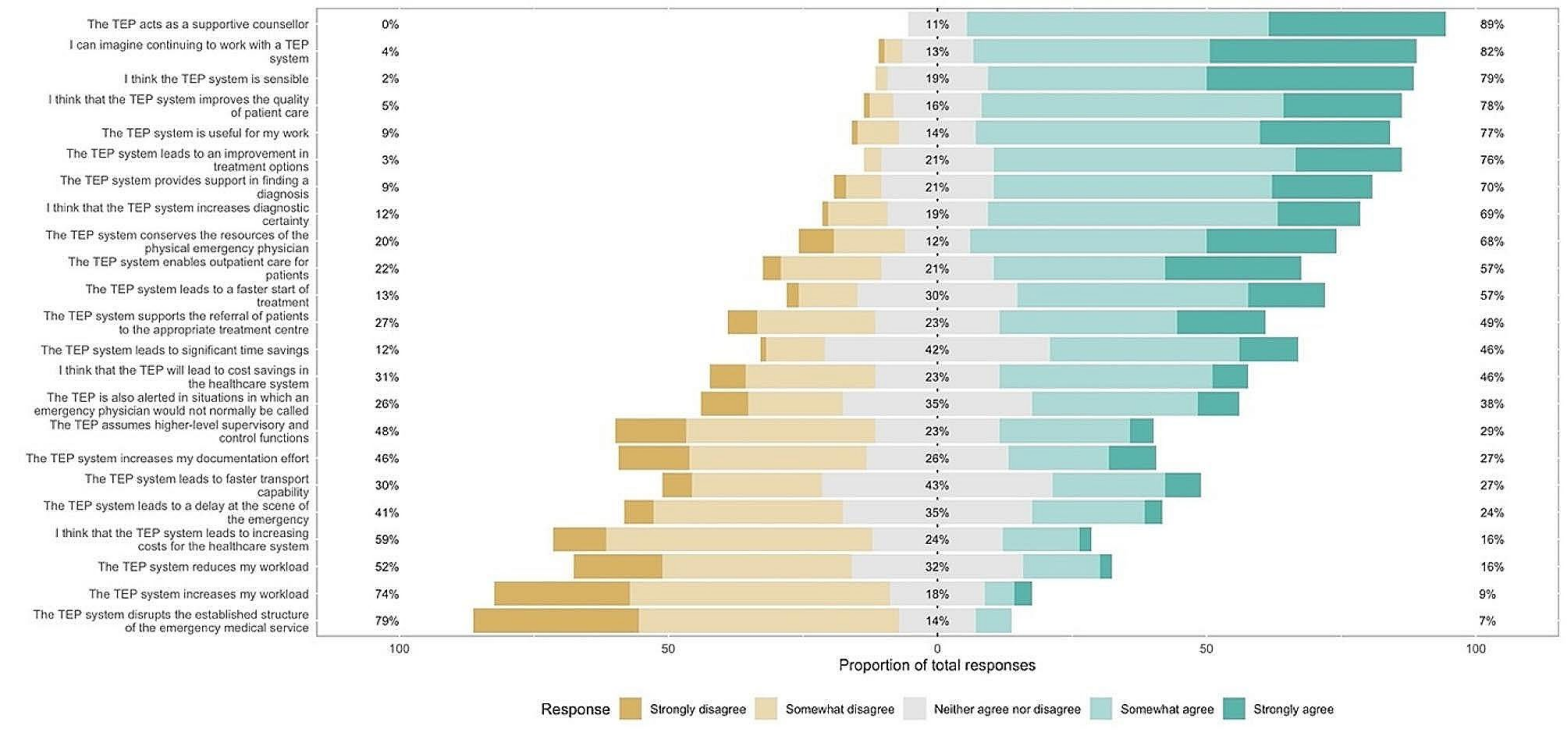
Visualization of the participants replies in proportion of agreement (green) and disagreement (yellow)

At the time of the study there were *n* = 308 registered professionals (including part-time employees and temporary staff), consisting of *n* = 238 (77,3%) non-physicians and *n* = 70 Emergency Physicians (22,7%). Of these *n* = 91 (29,5%) finished the complete survey and were included in the analysis (e.g. Figure 2).

The participants (*n* = 91) were on average 34,38 ± 10,89 years old 95% KI [33,27; 35,50]. The oldest being 59 and the youngest 19 years old.

Regarding sex *n* = 66 participants (72,55%) identified as male, while *n* = 25 (27,5%) as females.

*n* = 73 (80,2%) participants were qualified as emergency medical staff, which included paramedics *n* = 36 (39,56%), EMTs *n* = 28 (30,77%) and call handlers *n* = 9 (9,89%) for emergency dispatch centres. *n* = 18 (19,8%) were qualified as emergency physicians.

## Questionnaire

### Referral of patients to the appropriate treatment centre

A Mann-Whitney U test was performed to evaluate the item ”The telemedicine system supports the referral of patients to the appropriate treatment centre” between physician and non-physicians. Physicians significantly agreed with this item compared to non-physician medical staff [z=-2,074, *p* = .038].) The effect size was low *r*= 0.217.

A comparison within the group of non-physicians was also performed between EMTs (*n* = 24) 42,1% and Paramedics (*n* = 33) 57,9%.

48,5% of Paramedics and 33,3% of EMTs agreed with this item. The result was not significant [z=-1,845, *p* = .065].

### Level of knowledge about telemedicine

n = 43 (47,3%) Participants were assigned to the group “little or no knowledge”, n = 26 (28,6%) to the group “moderate level of knowledge” and n = 22 (24,2%) to group “high level of knowledge” group.

The item “The telemedical system leads to an improvement of treatment options” showed a significant difference [*χ*^2^ = 6,871, *p* = .032]. The Post-hoc test provided a significant difference between the groups “little or no knowledge” and “high level of knowledge” [z=-2,401, *p* = .049] as 90,3% of the “high level of knowledge” group agreed with this item compared to only 65,1% of the “little or no knowledge” group. The effect size was weak *r* = 0.295.

### Intended use of the telemedical system

36,4% of participants replied with a daily to weekly use from the group with a “high level of knowledge” compared to only 23,1% from the group with a “moderate level of knowledge” and 30,2% from the group “little or no knowledge”. There was no significant difference [*χ*^2^ = 9,521, *p* = .199] between the groups.

### Comparing age groups

Participants were assigned to 3 age groups: *n* = 42 (46,2%) participants were in the age group 19–31 years, *n* = 31 (34,1%) to the group 32–45 and *n* = 18 (19,8%) to the group 46–59 years.

The item “The tele-emergency physician performs higher-level supervisory and control functions” [*χ*^2^ = 12,958, *p* = .002] provided a significant group difference.

In the Post-hoc tests a significant group difference between the age groups “32–45” and “46–59” was seen [z=-3,356, *p* = .002] with a medium size of effect *r* = 0.479.

A significant difference was described for the age groups „19–31“ and „46–59“ [z=-3,205, *p* = .004] with a medium effect size *r* =0.413.

The participants from the groups 19–31 and 32–45 years agreed more with this item than the group of the 46–59 year olds.

### Frequency of intended use

The group „19–31“ years replied with 33,3% for a daily to weekly use, compared to 32,3% of the „32–45“ years group and 16,7% of the group „46–59“. There was no significant group difference [*χ*^2^ = 8,428, *p* = .215].

### Request for support

Regarding the items which asked the participants in which area a request for support is likely, the answer “decision on diagnosis and therapy” *n* = 71 (58,7%) was chosen the most frequently, followed by “organisational support” *n* = 24 (19,8%), “manual skills” *n* = 16 (13,2%), “no support needed” *n* = 4 (3,3%) and *n* = 6 (5,0%) for others (i.e. Table 1).

**Table 1:** Request for support per age group

Request for support		Age groups			
		**19–31** (*n* = 42)	**32–45** (*n* = 31)	**46–59** (*n* = 18)	**Overall** (*n* = 91)
Decision on diagnosis and therapy	*n*	*34*	*26*	*11*	71
	*% of group*	*81,0*	*83,9*	*61,1*	*77,17*
Organizational support	*n*	*9*	*4*	*11*	*24*
	*% of group*	*21,4*	*12,9*	*61,1*	*26,37*
Manual skills	*n*	*5*	*7*	*4*	*16*
	*% of group*	*11,9*	*22,6*	*22,2*	*17,58*
No support needed	*n*	*2*	*1*	*1*	*4*
	*% of group*	*4,8*	*3,2*	*5,6*	*4,4*

Comparing the 3 age groups *n* = 34 (81%) participants in the group 19–31 years and *n* = 26 (83,9%) from the group 32–45 requested support on “diagnosis and therapy”. Followed by “manual skills” *n* = 7 (22,6%) from the 32–45 years group and *n* = 4 (22,2%) from the group 46–59 years.

Regarding the qualification of participants, 75,8% of Paramedics (*n* = 25) and 95,8% of EMTs (*n* = 23) requested support on “diagnosis and therapy”.

## System usability scale

With Cronbachs Alpha being 0.829, overall “Usability” received a score of 68,68 (SD 12,76) 95% KI [67,37; 69,99] (i.e. Table 2).which allows a system to be described as usable [45].

**Table 2:** Usability evaluation with the System Usability Scale within the different groups

Categories		SUS Score			
		**n**	**mean**	**SD**	**Min - Max**
Age group	19–31 years	*42*	*69,46*	*14,7*	*37,5–100*
	32–45 years	*31*	*66,53*	*11,16*	*50–90*
	46–59 years	*18*	*70,56*	*10,38*	*50–92,5*
Qualification	Call handler	*9*	*68,89*	*11,87*	*57,5–92,5*
	Emergency Physician	*18*	*68,89*	*10,44*	*50–85*
	EMT	*28*	*66,07*	*14,73*	*37,5–92,5*
	Paramedic	*36*	*70,56*	*12,54*	*50–100*
Experience	< 2 years	*17*	*70,44*	*15,11*	*37,5–87,5*
	2–5 years	*17*	*65,59*	*11,27*	*42,5–87,5*
	6–10 years	*18*	*73,61*	*13,89*	*50–100*
	11–20 years	*24*	*66,25*	*11,37*	*47,5–92,5*
	21–30 years	*11*	*63,86*	*9,96*	*50–77,5*
	*> 31 years*	*4*	*80*	*7,36*	*70–87,5*

### Female and male participants

To compare the SUS Score in the female and male group a two-sided t-test was performed.

**Fig. 3:**
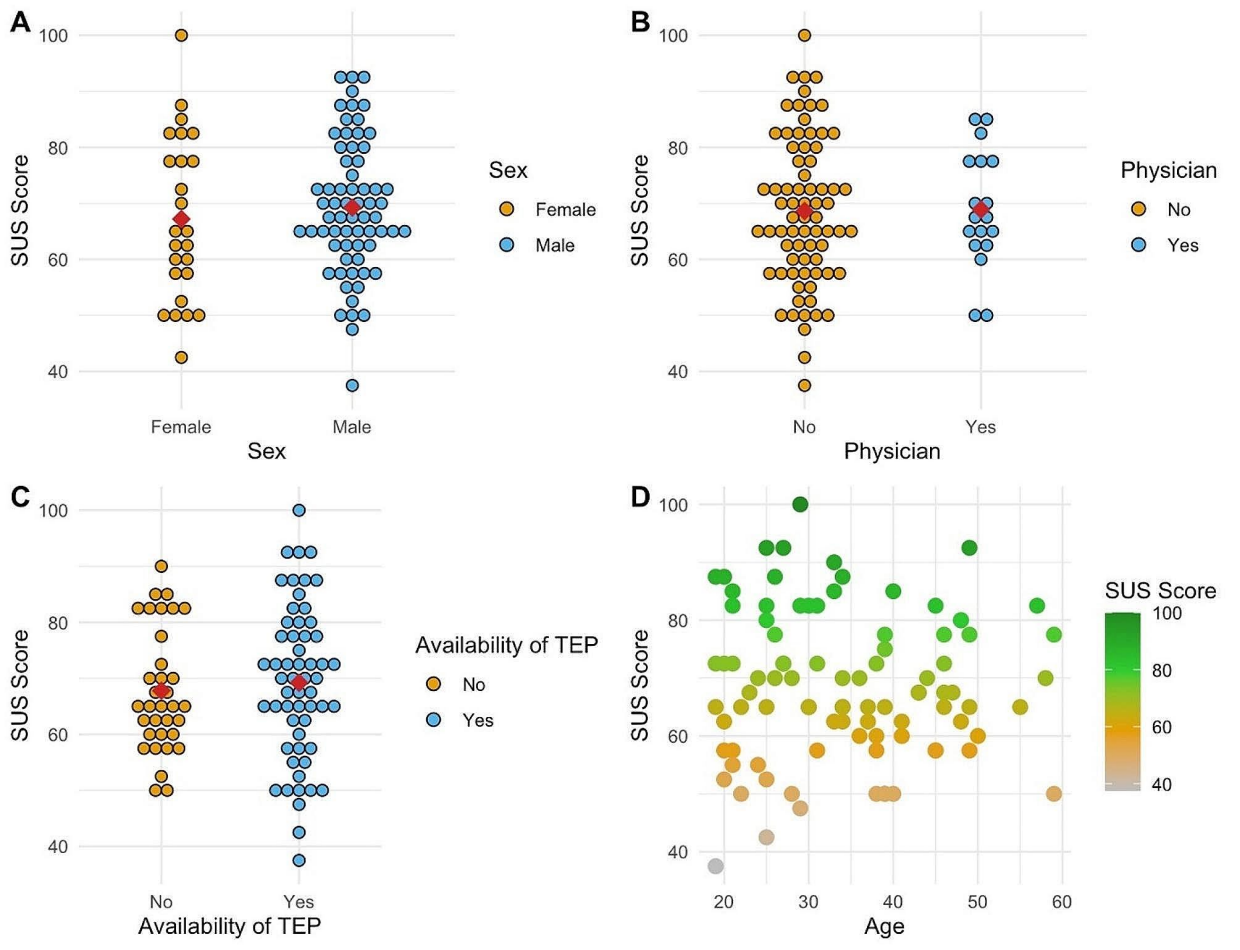
Evaluation of Usability results by the SUS Score differentiated for the categories: **A** - Sex, **B** - Physician vs. Non-Physician, **C** - Availability of TEP, **D** - Age

There was no significant difference in SUS Score between the male (M = 69.24, SD = 12, 95% KI [66.29; 72.18]) and female group (M = 67.20, SD = 14.74, 95% KI [61.12; 73.28]); [t(89) = 0.67952, *p* = .499]. (e.g. Fig. 3 Label A)

### Physicians and not physicians

There was no significant difference between the physician (M = 68.89, SD = 10.44, 95% KI [63.70; 74.08]) and non-physician group (M = 68.63, SD = 13.33, 95% KI [65.72; 71.94]) in SUS Scores [t(89) = 0.076628, *p* = .939]. (e.g. Fig. 3 Label B)

### Availability of TNA system

There was no significant difference in SUS Scores between the group that had a TNA System available (M = 69.21, SD = 13.82, 95% KI [65.54; 72.88]) and not-available (M = 67.79, SD = 10.9, 95% KI [65.72; 71.59]); [t(89) = 0.51014, *p* = .611] (e.g. Fig. 3 Label C).

### SUS and age

A Pearson correlation coefficient was computed to assess a linear relationship between the participants age and SUS score. There was a no correlation between the two variables, [r(89) =-0.002, *p* = .989] (e.g. Fig. 3 Label D).

### Results age groups

**Fig. 4:**
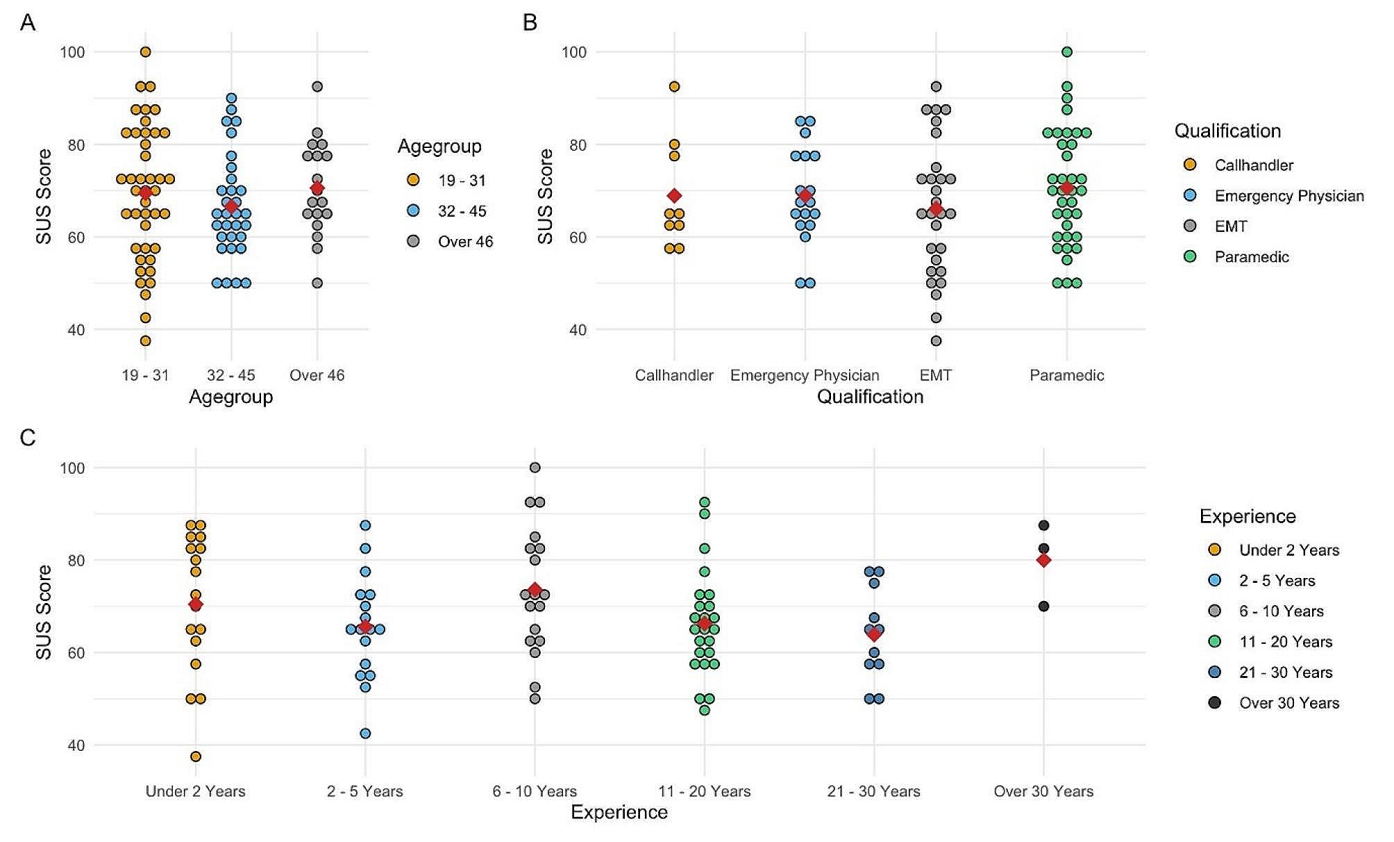
Evaluation of Usability results by the SUS Score differentiated for the categories: **A** - Agegroup, **B** - Qualification, **C** - Experience

A one-way ANOVA was performed to compare the effect of the age groups on the SUS Score. Homogeneity of variances was confirmed with Levene’s Test. It revealed that there was no statistically significant difference in mean SUS Scores between at least two groups [F(1, 89) = 0.038, *p* = .845]. (e.g. Fig. 4 Label A)

### Qualifications

When comparing the effects of the participants qualifications with the SUS Score, Levene’s Test confirmed homogeneity of variances. No statistically significant difference in mean SUS Scores between at least two groups [F(5, 85)=[2.028], *p* = .083] could be seen. (e.g. Fig. 4 Label B)

### Experience

When Comparing participants experience with the SUS Score, homogeneity of variances was confirmed with Levene’s Test but there was no statistically significant difference in between at least two groups [F(5, 85) = 2.029, *p* = .083]. (e.g. Fig. 4 Label C)

## Acceptability - usability - effectiveness

To explore the factorial structure 23 items (excluding sub-questions) were subjected to an exploratory factor analysis with orthogonal rotation. Further information on the Factor Analysis can be viewed in **Supplement 1**.

**Fig. 5:**
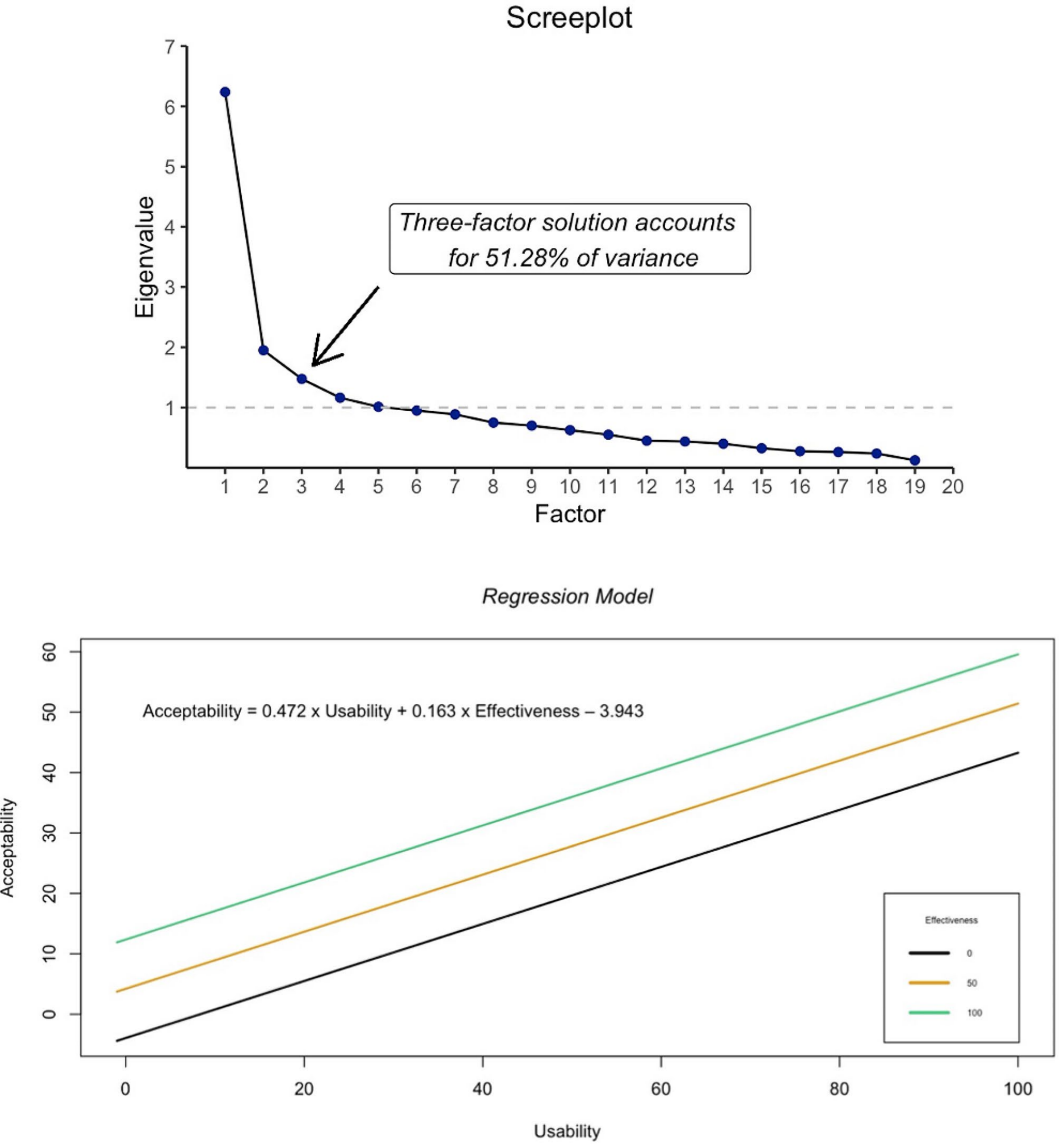
**Top**: Screeplot indicating that a three-factor solution accounts for the majority of variance. **Bottom**: Regression Model for “Acceptability” based on the variables “Usability” and “Effectiveness”

With the Kaiser’s criterion of eigenvalues greater than 1 and indicated by the scree plot a three-factor solution was yielded as the best fit for the data, accounting for 51.28% of variance (e.g. Figure 5). The results of this factor analysis are presented in detail in Supplement 1 including each Variable and MSA Value (e.g. Supplement 1).

As indicated by the scree plot, other possible factor solutions could be a 4- or 5-factor model (e.g. Fig. 5 Top). But such corresponding models don’t seem to exist in the available literature and marginally have a larger Eigenvalue than 1.

### Korrelation and Regression

The following reliability analysis provided that Cronbachs Alpha for the factor „Usability“ was 0.801, for „Effectiveness“ 0.779 and for the factor „Acceptably“ 0.805. All α-values were between 0.7 and 0.9, which describe consistent subscales [34].

A Pearson correlation coefficient was computed to assess the linear relationship between “Acceptability” and “Effectiveness”. Positive correlations between the two variables were seen, r(89) = .*439*, *p* < .001. and the size of effect was medium (i.e. Table 3).

**Table 3:** Correlations for acceptability, usability and effectiveness

	Acceptability	Usability
Usability	0.570*	
Effectiveness	0.439*	0.435*

* *p* < .001 ** *p* < .05

Between “Usability” and “Effectiveness” positive correlation existed r(89) = 0.435, *p* < .001 with a medium size effect (i. e. Table 3).

Also between “Acceptability” and “Usability” a positive correlation could be analysed r(89) = 0.570, *p* < .001. with a strong effect size (i.e. Table 3).

### Linear regression model

Based on the earlier results and on Sauers-Ford et al.’s concept, the factor “Acceptability” was analysed as a predictable variable and “Usability” and “Effectiveness” as predictor variables.

The regression model was: Acceptability = 0,472*Usability + 0,163*Effectiveness–3,943 (e.g. Fig. 5 Bottom).

Overall regression was statistically significant (R^2^ = 0.355, F(2, 88) = 25.801, *p* = .001).

It was found that “Usability” significantly predicted “Acceptability” (β = 0.467, *p* < .001) and “Effectiveness” significantly predicted “Acceptability” (β = 0.236, *p* = .014).

According to Cohen, the effect was strong: (𝑓2 = 0,55).

## Discussion

Successfully introducing and applying a telemedical system is challenging, but even more so in an interdisciplinary field like EM.

This is a challenge not only for those that are involved with the introduction of such a solutions but especially for those that will use these daily as well as those that manage clinical processes.

Therefore understanding the relevant factors of a successful technological roll-out during a continuous implementation to workflows becomes even more vital.

With our study we could not only confirm that – “Acceptability influences usability, which influences effectiveness” [28]– which already was described by Sauers-Ford et al., but could even quantify these effects: With “Usability” effecting “Acceptability” even more than “Effectiveness”, software and system developers but also those that are involved in choosing and implementing telemedical system need to focus more on “Usability”. Therefore choosing and introducing a system that offers a high level of “Usability” will increase its “Acceptability” far more than only focusing on a systems capability to solve a problem.

So if users perceive a system to be highly effective, it can still be that these will not accept the system, if the perceived “Usability” is not adequate. In worst case the system would not be used and the misinvested funds could have been applied elsewhere in a healthcare system that already struggles with increasingly tighter.

Therefore our results could not only extend this principle but even emphasize its relevance for the whole field of EM as three factors represent over 51% of the influencing effects.

Therefore we recommend that regulators and administrators should perform such analysis regularly and not only monitor existing introduction processes. Furthermore the impact of trainings but also system updates need to be recognized, to allow an earlier handling of problems or recurring challenges.

With biases and beliefs being a relevant factor in accepting changes in one’s work environment, understanding these becomes even more relevant:

Overall a positive attitude could be attest to the participants as telemedicine is seen as an advantageous tool to generally improve treatment options and processes. Especially those that have access to a telemedical system see that there are more treatment options available.

Regarding the introduction within established processes, the results showed a small tendency to see the TEP System to increase general workload.

This proves even more that such systems need to focus not only on being effective but on being usable. This includes rapid availability, improved “Acceptability” and adaptability for various scenarios. Ideally within an established technological ecosystem to improve its effectivity.

While physician participants agreed more with the item that a TEP system allows the referral of patients to an appropriate treatment centre so that specialists can be rapidly available at the site of emergency, this availability could improve patients quality of treatment and also reduce unnecessary transfers [46]. While this advantage seems to be a present thought for the participating physicians, further extended education will be needed for paramedics. As a new communication network could allow more treatment options, the overall processes in the pre-clinical field will change and would involve paramedics to perform f.ex. more advanced treatments while being supervised by the TEP.

Most participants agreed that a decision on diagnosis and therapy would be the most common request for support currently. With this being a common theme in many surveys, even some which have been published a decade ago [15, 36], this reply provides a relevant insight as current telemedical systems - especially in Germany- focus on only being a tool to replace emergency physicians. With a further development of telemedical solutions such systems will probably not only be used for communication on treatment and diagnosis, but will also need to be able to allow a broader application. For example a support on manual skills could be performed if technologies like Augmented Reality (AR) and Point-of-Care Ultrasound could be combined, while allowing supervision by a TEP.

Developing a broad network solution –like an emergency talk network [22]- could therefore allow not only specialists to be available rapidly but allow advanced imaging to be used for diagnosis and treatment. Combining advanced technology with advanced treatment possibilities in the pre-hospital field could be one of many options.

With an overall “Usability” rating of 68,68 this system would be described as usable, but at a marginal range [47]. In an early stage of introduction such an evaluation could be expected, but a continuous focus on “Usability” is needed if the long-term goal is high “Acceptability”.

Furthering education and advancing available training could improve implementation while the effects shoud be monitored. At the same time optimising the system according to human factor design and user recommendations [27] would be the most promising approach for an improvement in telemedical systems.

## Limitations

As this trial was only performed in one region of Germany and only involved one telemedical system a direct generalizability of our results could only be performed with regards to these limitations.

EM systems vary nationally and some regions only involve emergency physician for special circumstances. Systems that are developed and used in these countries will need different specifications regarding the user’s needs.

As the sample size especially for a comparison of different groups and specifications were rather small a generalization can also only be performed with limited applicability.

With this being the first reported factor analysis, further confirmatory analysis should be performed in EM. Especially understanding the other involved factors as the effect of the remaining 48% are still unclear. Therefore further research is needed to understand this large proportion as not only implementation processes but also the development of better telemedical systems could allow an improved and more individualised application of telemedicine in prehospital EM.

## Conclusions

When introducing a telemedical system a deep understanding of the involved structures, legislation, medical cases, regional differences but especially the users is required. Developing an understanding of these effects is relevant and requires a framework to improve the implementation process. Therefore quantifying this process allows decision makers to understand the challenges and which steps can provide the most impact. Focusing on aspects of “Usability” will improve the acceptance of systems even more than aspects that only focus on a systems “Effectiveness”.

With our approach we developed a framework which can be applied to various settings of telemedicine in EM. But this framework needs further testing and validation in other settings. Telemedicine will not only be a technology to replace currently missing staff or resources but will be a technology that will add more treatment options at the site of emergency in combination with novel developments.

### Electronic supplementary material

Below is the link to the electronic supplementary material.

Supplementary Material 1

Supplementary Material 2

## Data Availability

Data is provided within the manuscript or supplementary information files. The datasets used and/or analysed during the current study are available from the corresponding author on reasonable request.
