# Supplementary material for: Improving the introduction of telemedicine in pre-hospital emergency medicine: understanding users and how acceptability, usability and effectiveness influence this process
Source: BMC Emerg Med. 2024 Jul 12;24:114. doi: 10.1186/s12873-024-01034-6 (PMC11241972; doi:10.1186/s12873-024-01034-6)
Supplement: Supplementary file 1 — Supplementary Material 1 [file 12873_2024_1034_MOESM1_ESM.pdf]

Anhang

Fragebogen

LimeSurvey

Liebe Teilnehmerin, lieber Teilnehmer,

mein Name ist Jennifer Krautwald und ich bin Studentin des Masterstudiengangs Public Health an der Technischen Hochschule Mittelhessen. Im Rahmen meiner Abschlussarbeit führe ich, gemeinsam mit der AG Technologien in der Notfallmedizin, eine wissenschaftliche Erhebung über die Potenziale und Herausforderungen eines präklinischen Telenotarztsystems durch.

Ziel ist es, Handlungsempfehlungen für eine optimale Nutzung von TNA-Systemen abzuleiten, um diese auch zukünftig wirksam einzusetzen und bestmöglich in der Regelversorgung zu etablieren.

Die Teilnahme an der Befragung ist freiwillig, kann aber nur verlässliche Ergebnisse liefern, wenn möglichst viele unterschiedliche Personen daran teilnehmen. Daher bin ich Ihnen sehr dankbar, wenn Sie die Gelegenheit nutzen, um Ihre Ansichten und Erfahrungen zu teilen und in das Ergebnis der Studie einfließen zu lassen.

Für den Erfolg dieser Studie ist es wichtig, dass der Fragebogen vollständig und wahrheitsgemäß ausgefüllt wird. Die ungefähre Bearbeitungszeit dieses Fragebogens beträgt ca. 10 Minuten.

Alle Daten werden anonym erhoben, können Ihrer Person nicht zugeordnet werden und werden streng vertraulich behandelt.

Die Daten werden nur im Rahmen dieser Untersuchung gespeichert und der Zugang ist nur den Datenerhebern der Technischen Hochschule Mittelhessen möglich. Die Daten werden nur für wissenschaftliche Zwecke genutzt und ggf. anonymisiert bei wissenschaftlichen Fachkongressen bereitgestellt.

Bei Fragen oder Anregungen stehen wir Ihnen gerne zur Verfügung:

jennifer.krautwald@ges.thm.de

sean.o.sullivan@ges.thm.de

(Seán O'Sullivan - AG Technologien in der Notfallmedizin)

Ich danke Ihnen für Ihre Zeit und die Teilnahme an dieser Befragung!

Jennifer Krautwald

AG Technologien in der Notfallmedizin

Technische Hochschule Mittelhessen

Fachbereich Gesundheit

Wiesenstraße 14

35390 Gießen

Abbildung 19: Fragebogen Seite 1

LimeSurvey

Teil A: Allgemeiner Part

Zunächst möchte ich Sie um einige Angaben zu Ihrer Person, Ihrer beruflichen Position und der Organisation, bei der Sie aktuell beschäftigt sind, bitten.

A1. Wie alt sind Sie?

A2. Welches Geschlecht haben Sie?

- Männlich ☐
- Weiblich ☐
- Divers ☐
- Keine Angabe ☐

A3. In welcher Art von Organisation arbeiten Sie im Rahmen der Notfallversorgung überwiegend?

- Leitstelle ☐
- Rettungsdienst ☐
- Klinik ☐
- Sonstiges ☐

Sonstiges

A4. In welcher Position arbeiten Sie aktuell?

- (Not-) Arzt/Ärztin ☐
- Notfallsanitäter*in ☐
- Rettungsassistent*in ☐
- Rettungssanitäter*in ☐
- Leitstellendisponent*in ☐
- Sonstiges ☐

Sonstiges

A5. Wie lange führen Sie diesen Beruf (unabhängig vom Arbeitgeber) bereits aus?

- Unter 2 Jahre ☐
- 2 - 5 Jahre ☐
- 6 - 10 Jahre ☐
- 11 - 20 Jahre ☐
- 21 - 30 Jahre ☐
- Über 30 Jahre ☐

Teil B: Telenotarztsystem

Im Folgenden finden Sie einige Aussagen in Bezug auf Ihre berufliche Situation und in Bezug auf das Telenotarztsystem.

B1. Auf einer Skala von 1 bis 10: Wie stark trifft die folgende Aussage auf Sie zu?

Ich erlebe Situationen, in denen ich mir Unterstützung durch einen erfahrenen Kollegen wünsche.

1 - Nie	2	3	4	5	6	7	8	9	10 - Immer
☐	☐	☐	☐	☐	☐	☐	☐	☐	☐

Abbildung 20: Fragebogen Seite 2

LimeSurvey

B2. Dabei wünsche ich mir vor allem Unterstützung bei...

(Mehrfachnennung möglich)

- ☐ Manuellen Tätigkeiten
- ☐ Diagnosefindung und Therapieentscheidung
- ☐ Organisatorischen Tätigkeiten
- ☐ Ich wünsche mir keine Unterstützung
- ☐ Neben den oben genannten Angaben wünsche ich mir außerdem Unterstützung bei:

Neben den oben genannten Angaben wünsche ich mir außerdem Unterstützung bei:

B3. Besteht in Ihrem Arbeitsbereich die Möglichkeit, ein Telenotarztsystem zu nutzen?

- ☐ Ja
- ☐ Nein

B4. Auf einer Skala von 1 bis 10: Wie bewerten Sie Ihren derzeitigen Kenntnisstand in Bezug auf das Telenotarztsystem?

1 - Sehr gering	2	3	4	5	6	7	8	9	10 - Sehr hoch
☐	☐	☐	☐	☐	☐	☐	☐	☐	☐

B5. Wie oft verwenden Sie das Telenotarztsystem?

- ☐ Täglich
- ☐ Mindestens ein Mal wöchentlich
- ☐ Mindestens ein Mal in zwei Wochen
- ☐ Mindestens ein Mal monatlich
- ☐ Seltener als ein Mal monatlich
- ☐ Nie

B6. Aus welchen Gründen verwenden Sie das Telenotarztsystem seltener als ein Mal monatlich?

B7. Aus welchen Gründen verwenden Sie das Telenotarztsystem nie?

Abbildung 21: Fragebogen Seite 3

LimeSurvey

B8. Wie stark stimmen Sie den folgenden Aussagen zu?

Bitte bewerten Sie die folgenden Aussagen hinsichtlich Ihrer Erwartungen oder Ihrer bisherigen Erfahrungen mit dem Telenotarztsystem.

(Falls diese Umfrage auf einem Smartphone aufgerufen wird, erfolgt bei Bedarf eine vereinfachte Darstellung der nachfolgenden Aussagen, wenn Sie das Smartphone im Querformat nutzen.)

	Stimme überhaupt nicht zu	Stimme eher nicht zu	Teils / teils	Stimme eher zu	Stimme voll zu
Ich halte das Telenotarztsystem für sinnvoll.	☐	☐	☐	☐	☐
Das Telenotarztsystem führt zu einer relevanten Zeitersparnis.	☐	☐	☐	☐	☐
Das Telenotarztsystem erhöht meine Arbeitsbelastung.	☐	☐	☐	☐	☐
Das Telenotarztsystem unterstützt bei der Diagnosefindung.	☐	☐	☐	☐	☐
Das Telenotarztsystem führt zu einem schnelleren Therapiebeginn.	☐	☐	☐	☐	☐
Das Telenotarztsystem führt zu einer schnelleren Transportfähigkeit.	☐	☐	☐	☐	☐
Das Telenotarztsystem führt zu einer Verzögerung am Notfallort.	☐	☐	☐	☐	☐
Ich denke, dass das Telenotarztsystem die Qualität der Patientenversorgung verbessert.	☐	☐	☐	☐	☐
Das Telenotarztsystem führt zu einer Verbesserung der Behandlungsmöglichkeiten.	☐	☐	☐	☐	☐
Ich denke, dass das Telenotarztsystem die Diagnosesicherheit erhöht.	☐	☐	☐	☐	☐
Das Telenotarztsystem unterstützt bei der Zuweisung von Patienten in die geeignete Behandlungseinrichtung.	☐	☐	☐	☐	☐
Das Telenotarztsystem ermöglicht die ambulante Versorgung von Patienten.	☐	☐	☐	☐	☐
Das Telenotarztsystem erhöht meinen Dokumentationsaufwand.	☐	☐	☐	☐	☐
Das Telenotarztsystem ist nützlich für meine Arbeit.	☐	☐	☐	☐	☐
Das Telenotarztsystem vermindert meine Arbeitsbelastung.	☐	☐	☐	☐	☐
Der Telenotarzt übernimmt die Funktion eines unterstützenden Beraters.	☐	☐	☐	☐	☐
Der Telenotarzt übernimmt übergeordnete Aufseher- und Kontrollfunktionen.	☐	☐	☐	☐	☐
Der Telenotarzt wird auch in Situationen alarmiert, in denen normalerweise kein Notarzt alarmiert wird.	☐	☐	☐	☐	☐
Das Telenotarztsystem stört die etablierte Struktur des Rettungsdienstes.	☐	☐	☐	☐	☐
Durch das Telenotarztsystem wird die Ressource des physischen Notarztes geschont.	☐	☐	☐	☐	☐
Ich denke, dass das Telenotarztsystem zu Kosteneinsparungen im Gesundheitswesen führt.	☐	☐	☐	☐	☐
Ich denke, dass das Telenotarztsystem zu Kostensteigerungen im Gesundheitswesen führt.	☐	☐	☐	☐	☐
Ich kann mir vorstellen, weiterhin mit einem Telenotarztsystem zu arbeiten.	☐	☐	☐	☐	☐

B9. Wie häufig beabsichtigen Sie, die Telenotarztanwendung zukünftig bei geeigneten Einsätzen zu benutzen?

- Täglich ☐
- Mindestens ein Mal wöchentlich ☐
- Mindestens ein Mal in zwei Wochen ☐
- Mindestens ein Mal monatlich ☐
- Seltener als ein Mal monatlich ☐
- Nie ☐

Abbildung 22: Fragebogen Seite 4

LimeSurvey

Teil C: Usability des Telenotarztsystems

Im Folgenden finden Sie Aussagen zur Benutzerfreundlichkeit bzw. Gebrauchstauglichkeit des Telenotarztsystems.

C1. **Wie stark stimmen Sie den folgenden Aussagen zu?**

Bitte bewerten Sie die folgenden Aussagen hinsichtlich Ihrer Erwartungen oder Ihrer bisherigen Erfahrungen mit dem Telenotarztsystem.

(Falls diese Umfrage auf einem Smartphone aufgerufen wird, erfolgt bei Bedarf eine vereinfachte Darstellung der nachfolgenden Aussagen, wenn Sie das Smartphone im Querformat nutzen.)

	1 - Stimme überhaupt nicht zu	2	3	4	5 - Stimme voll zu
Ich denke, dass ich das System gerne häufig benutzen würde.	☐	☐	☐	☐	☐
Ich finde das System unnötig komplex.	☐	☐	☐	☐	☐
Ich finde das System einfach zu benutzen.	☐	☐	☐	☐	☐
Ich benötige die Hilfe einer technisch versierten Person, um das System benutzen zu können.	☐	☐	☐	☐	☐
Ich finde, die verschiedenen Funktionen in diesem System sind gut integriert.	☐	☐	☐	☐	☐
Ich denke, das System enthält zu viele Inkonsistenzen.	☐	☐	☐	☐	☐
Ich kann mir vorstellen, dass die meisten Menschen den Umgang mit diesem System sehr schnell lernen.	☐	☐	☐	☐	☐
Ich finde das System sehr umständlich zu nutzen.	☐	☐	☐	☐	☐
Ich fühle mich bei der Benutzung des Systems sehr sicher.	☐	☐	☐	☐	☐
Ich musste eine Menge lernen, bevor ich anfangen konnte das System zu verwenden.	☐	☐	☐	☐	☐

Teil D: Telenotarztsystem - offene Fragen

Im letzten Abschnitt finden Sie sechs offene Fragen in Bezug auf das Telenotarztsystem, deren Beantwortung freiwillig ist.

Ich bin Ihnen sehr dankbar, wenn Sie die Gelegenheit nutzen, hier Ihre Kenntnisse, Erfahrungen und Wünsche zu teilen.

D1. **Wenn Sie an Ihre letzte Nutzung des Telenotarztsystems denken: Was hat gut funktioniert?**

D2. **Was würden Sie bei Ihrer nächsten Nutzung anders machen?**

D3. **Wenn Sie Änderungen am Telenotarztsystem oder -prozess vornehmen könnten, welche System- oder Prozessänderungen würden Sie vornehmen und warum?**

D4. **Wie entscheiden Sie, ob Sie das Telenotarztsystem nutzen? Welche Diagnosen oder Probleme sind für Sie ausschlaggebend?**

D5. **Was erhoffen und wünschen Sie sich von der hessenweiten Einführung einer Telenotarztanwendung?**

D6. **Welche negativen Konsequenzen befürchten Sie durch die hessenweite Einführung einer Telenotarztanwendung?**

Abbildung 23: Fragebogen Seite 5

LimeSurvey

Vielen Dank für Ihre Teilnahme an dieser Umfrage!

Bei Rückfragen stehen wir Ihnen gerne zur Verfügung:

jennifer.krautwald@ges.thm.de

sean.o.sullivan@ges.thm.de

Ich bedanke mich für Ihre Zeit,

Jennifer Krautwald

AG Technologien in der Notfallmedizin

Technische Hochschule Mittelhessen

Fachbereich Gesundheit

Wiesenstraße 14

35390 Gießen

Abbildung 24: Fragebogen Seite 6

Original Fragen und Vorgenommene Änderungen des Fragebogens / Original Items and Changes (Deutsch / Englisch)

**Originalfrage (German)**	**Änderung / *Adapation***	**Grund / *Reason***	**Final English Version**
**Teil 1 – Allgemeiner Part**			
---	Wie alt sind Sie? / *How old are you?*	Die Frage wurde in den Fragebogen aufgenommen, um altersspezifische Vergleiche zu ermöglichen. / *The question was included in the questionnaire to enable age-specific comparisons.*	How old are you?
Wie lange sind Sie bereits bei Ihrer derzeitigen Organisation beschäftigt?	Entfernt / *Removed*	Die Frage wurde nach Auswertung des Pretests entfernt, da auf Grundlage dieser Frage keine Berechnungen durchgeführt wurden. / *The question was removed after the pre-test was analysed, as no calculations were carried out on the basis of this question.*	---
**Teil 2 – Notfallmedizin & Telenotarztsystem (Likert-Skala)**			
Ich fühle mich in der Notfallmedizin kompetent	Entfernt / *Removed*	Die betreffenden Aussagen wurden vor Durchführung des Pretests entfernt, da auf Grundlage dieser Aussagen keine Berechnungen durchgeführt werden sollten. / *The question was removed after the pre-test was analysed, as no calculations were carried out on the basis of this question.*	---
Bei der Auskultation der Lunge erkenne ich… (Seitengleiche Belüftung / Giemen und Brummen / einen Pneumothorax)			---
Ein 12-Kanal-EKG kann ich… (Schreiben / Auswerten / einem Kollegen erklären)			---
Risiken und Nebenwirkungen von Opiaten kann ich… (Nennen / Erkennen / Behandeln)			---

Eine Hypoglykämie kann ich… (Erkennen / Therapieren / Therapieren und die Pathophysiologie dahinter erkennen)			---
Als Atemswegsicherung traue ich mir zu… (Maskenbeatmung / Endotracheale Intubation / Platzieren einer Larynxmaske)			---
Der Umgang mit modernen Kommunikationsmitteln fällt mir leicht.			---
Modernen Kommunikationsmitteln stehe ich privat aufgeschlossen gegenüber.			---
Beruflich arbeite ich viel mit modernen Kommunikationsmitteln.			---
Ich finde Zusatzfunktionen im Internet, wie z.B. Rote Liste, Giftnotrufzentrale, Leitlinien, hilfreich.			---
Ich nutze regelmäßig Zusatzfunktionen im Internet, wie z.B. Rote Liste, Giftnotrufzentrale, Leitlinien.			---
Haben Sie bereits bei telemedizinischen Projekten mitgemacht?			---

Kannten Sie die Telenotarztanwendung bereits vor der heutigen Befragung?			---
Ich erlebe Situationen, in denen ich mir Unterstützung durch einen erfahrenen Kollegen wünsche.	Änderung der Antwortoptionen von „Selten – Häufig“ in eine numerische Skala von „1 (nie) – 10 (immer)“ / *Change the answer options from "Rarely - Often" to a numerical scale of "1 (never) - 10 (always)" /*	Die Änderung wurde nach Auswertung des Pretests vorgenommen, um den Interpretationsspielraum zu reduzieren, indem nun numerische Werte verwendet werden. Die ursprünglichen Antwortoptionen „selten“ und „häufig“ basieren auf subjektiven und individuellen Empfindungen, was zu potenziellen Verzerrungen in den Ergebnissen hätte führen können. Die Verwendung von numerischen Werten ermöglicht eine objektivere und präzisere Messung, wodurch eine bessere Vergleichbarkeit der Antworten erzielt wird. // *The change was made after analysing the pre-test in order to reduce the scope for interpretation by now using numerical values. The original response options "rarely" and "frequently" were based on subjective and individual perceptions, which could have led to potential distortions in the results. The use of numerical values allows for a more objective and precise measurement, resulting in better comparability of responses.*	On a scale of 1 to 10: How strongly does the following statement apply to you? I experience situations in which I would like support from an experienced colleague.
---	Besteht in Ihrem Arbeitsbereich die Möglichkeit, ein Telenotarztsystem zu nutzen?/ *New Question added: Is it possible to use a tele-emergency physician system in your area of work?*	Die Frage wurde in den Fragebogen aufgenommen, da nicht alle Rettungsdienstbereiche des MTK über ein Telenotarztsystem verfügen. / *The question was included in the questionnaire because not all ambulances in MTK have a tele-emergency physician system*.	Is it possible to use a tele-emergency physician system in your area of work?
---	Auf einer Skala von 1 bis 10: Wie bewerten Sie Ihren derzeitigen Kenntnisstand in Bezug auf das Telenotarztsystem? / *On a scale of 1 to 10, how would you rate your current level of knowledge of the tele-emergency physician system?*	Die Frage wurde in den Fragebogen aufgenommen, um gruppenspezifische Vergleiche hinsichtlich des Kenntnisstands zu ermöglichen. / *The question was included in the questionnaire to enable group-specific comparisons with regard to the level of knowledge.*	On a scale of 1 to 10, how would you rate your current level of knowledge of the tele-emergency physician system?

---	Wie oft verwenden Sie das Telenotarztsystem? / *How often do you use the telemedicine system?*	Die betreffende Frage wurde nach Rücksprache mit den ÄLRDs in den Fragebogen aufgenommen. / *The question was included in the questionnaire at the request of the ÄLRD..*	How often do you use the telemedicine system?
Mir vorgesetzte Personen halten das Konzept des Telenotarztes für sinnvoll.	Entfernt / *Removed*	Die betreffenden Aussagen wurden vor der Durchführung des Pretests entfernt, da das Hauptinteresse darin lag, zu ermitteln, ob die befragte Person das Telenotarztsystem als sinnvoll erachtet. Auf Basis dieser Einschätzungen wurden entsprechende Berechnungen durchgeführt. Die Entfernung dieser Aussagen ermöglichte somit eine gezieltere und fokussierte Datenerhebung, die auf die spezifische Fragestellung ausgerichtet war. / *The statements in question were removed before the pre-test was carried out, as the main interest was to determine whether the interviewee considered the telemedicine system to be useful. Corresponding calculations were carried out on the basis of these assessments. The removal of these statements thus enabled a more targeted and focussed data collection that was geared towards the specific question.*	---
Kollegen, die mir wichtig sind, halten das Konzept des Telenotarztes für sinnvoll.			---
Personen in meinem Freundeskreis halten das Konzept des Telenotarztes für sinnvoll.			---
Wenn ich in einem Projekt, wie dem Telenotarzt, mitmache, steigt mein Ansehen im Kollegenkreis.		Die betreffenden Aussagen wurden vor der Durchführung des Pretests entfernt, da das Ansehen und die Akzeptanz von außen für das Studienziel keine relevanten Einflussfaktoren darstellen. / *The statements in question*	---

Wenn ich in einem Projekt, wie dem Telenotarzt, mitmache, steigt mein Ansehen im Freundeskreis.		*were removed before the pre-test was carried out, as external reputation and acceptance are not relevant influencing factors for the study objective.*	
Ich denke, dass das Konzept des Telenotarztes zu einer relevanten Zeitersparnis führt.	Das Telenotarztsystem führt zu einer relevanten Zeitersparnis. / *The tele-emergency physician system leads to significant time savings.*	Die entsprechenden Änderungen wurden vor der Durchführung des Pretests vorgenommen. Dabei wurden die Wörter „Ich denke“ entfernt, da das Telenotarztsystem bereits in zwei Rettungsdienstbereichen des MTK genutzt wird. Somit werden keine Vermutungen abgefragt, sondern die tatsächlichen Einstellungen und Meinungen der Teilnehmer erfasst. Zudem handelt es sich nicht um das „Konzept des Telenotarztes“, sondern um das „Telenotarztsystem“ im MTK. Durch diese präzise Formulierung wird sichergestellt, dass die Aussagen den tatsächlichen Erfahrungen und Einschätzungen der Teilnehmer entsprechen und keine Verständnisprobleme auftreten. / *The corresponding changes were made before the pre-test was carried out. The words "I think" were removed, as the tele-emergency physican system was already being started to be used in two ambulance service areas of the MTK. This means that no assumptions are asked, but the actual attitudes and opinions of the participants are recorded. Furthermore, it is not about the "concept of the tele-EMS physician", but about the "tele-EMS physician system" in the MTK. This precise formulation ensures that the statements correspond to the actual experiences and assessments of the participants and that no problems of understanding arise.*	The tele-emergency physician system leads to significant time savings.
Ich denke, dass das Konzept des Telenotarztes zu einer schnelleren Diagnosefindung führt.	Das Telenotarztsystem unterstützt bei der Diagnosefindung./ The tele-emergency physician system provides support in finding a diagnosis.		The tele-emergency physician system provides support in finding a diagnosis.
Ich denke, dass das Konzept des Telenotarztes zu einem schnelleren Therapiebeginn führt.	Das Telenotarztsystem führt zu einem schnelleren Therapiebeginn. / *The tele-emergency physician system leads to a faster start of treatment.*		The tele-emergency physician system leads to a faster start of treatment.
Ich denke, dass das Konzept des Telenotarztes zu einer schnelleren Transportfähigkeit führt.	Das Telenotarztsystem führt zu einer schnelleren Transportfähigkeit. / *The tele-emergency physician system leads to faster transport capability.*		The tele-emergency physician system leads to faster transport capability.
Ich denke, dass das Konzept des Telenotarztes zu einer Verzögerung am Notfallort führt.	Das Telenotarztsystem führt zu einer Verzögerung am Notfallort. / *The tele-emergency physician system leads to a delay at the scene of the emergency.*		The tele-emergency physician system leads to a delay at the scene of the emergency.
Ich denke, dass das Konzept des Telenotarztes nützlich für meine Arbeit ist.	Das Telenotarztsystem ist nützlich für meine Arbeit. / *The TEP system is useful for my work.*		The TEP system is useful for my work.

Ich denke, dass das Konzept des Telenotarztes meine Arbeitsbelastung vermindert.	Das Telenotarztsystem vermindert meine Arbeitsbelastung. / *The TEP system reduces my workload.*		The TEP system reduces my workload.
Ich denke, dass das Konzept des Telenotarztes die etablierte Struktur stört.	Das Telenotarztsystem stört die etablierte Struktur des Rettungsdienstes. / *The TEP system disrupts the established structure of the emergency medical service.*		The TEP system disrupts the established structure of the emergency medical service.
---	Das Telenotarztsystem erhöht meine Arbeitsbelastung. / *The tele-emergency physician system increases my workload.*	Vor der Durchführung des Pretests wurde eine Kontrollfrage in den Fragebogen aufgenommen. Dadurch wurde die Anzahl der negativen Aussagen erhöht, um eine Risikobewertung des Telenotarztsystems zu ermöglichen. Die Formulierung der Aussage wurde aus der Studie von Kuntosch et al. entnommen, was zudem eine Vergleichbarkeit der Ergebnisse ermöglicht. / *A control question was included in the questionnaire before the pre-test was carried out. This increased the number of negative statements in order to enable a risk assessment of the telemedicine system. The wording of the statement was taken from the study by Kuntosch et al., which also allows the results to be compared*	The tele-emergency physician system increases my workload.
---	Das Telenotarztsystem führt zu einer Verbesserung der Behandlungsmöglichkeiten. / *The TEP system leads to an improvement in treatment options.*	Die betreffenden Aussagen wurden auf Wunsch des ÄLRD vor der Durchführung des Pretests in den Fragebogen aufgenommen. Die Einbeziehung dieser Aussagen erfolgte, um den Fragebogen gemäß den Anforderungen und Erkenntnissen anzupassen und relevante Aspekte für die Studie zu berücksichtigen. / *The relevant statements were included in the questionnaire at the request of the ÄLRD before the pre-test was carried out. These statements were included in order to adapt the questionnaire to the*	The TEP system leads to an improvement in treatment options.
---	Ich denke, dass das Telenotarztsystem die Diagnosesicherheit erhöht. / *I think that the TEP system increases diagnostic certainty.*		I think that the TEP system increases diagnostic certainty.

---	Das Telenotarztsystem unterstützt bei der Zuweisung von Patienten in die geeignete Behandlungseinrichtung. / *The TEP system supports the referral of patients to the appropriate treatment centre.*	*requirements and findings of the ÄLRD and to take into account relevant aspects for the study.*	The TEP system supports the referral of patients to the appropriate treatment centre.
---	Das Telenotarztsystem ermöglicht die ambulante Versorgung von Patienten. / *The TEP system enables outpatient care for patients.*		The TEP system enables outpatient care for patients.
Ich denke, dass das Konzept des Telenotarztes meinen Dokumentationsaufwand mindert.	Das Telenotarztsystem erhöht meinen Dokumentationsaufwand. / *The TEP system increases my documentation effort*.	Vor der Durchführung des Pretests wurde eine weitere negative Aussage in den Fragebogen aufgenommen. Dadurch wurde die Anzahl der negativen Aussagen erhöht, um eine Risikobewertung des Telenotarztsystems zu ermöglichen. Die Formulierung der Aussage wurde aus der Studie von Kuntosch et al. entnommen, was zudem eine Vergleichbarkeit der Ergebnisse ermöglicht. / Before the pre-test was carried out, a further negative statement was included in the questionnaire. This increased the number of negative statements in order to enable a risk assessment of the telemedicine system. The wording of the statement was taken from the Kuntosch et al. study, which also allows the results to be more comparable.	The TEP system increases my documentation effort.
---	Der Telenotarzt übernimmt die Funktion eines unterstützenden Beraters. / The TEP acts as a supportive counsellor.	Die Aussagen wurden aufgrund des Hinweises des ÄRLD der am Pretest teilnahm, in den Fragebogen aufgenommen. / The statements were included in the questionnaire based on the advice of the ÄLRD.	The TEP acts as a supportive counsellor.
---	Der Telenotarzt übernimmt übergeordnete Aufseher- und Kontrollfunktionen. / *The TEP assumes higher-level supervisory and control functions.*		The TEP assumes higher-level supervisory and control functions.

<table>
<tr><td>Ich vermute, der fehlende Tastsinn wird die Arbeit des Telenotarztes beeinträchtigen.</td><td rowspan="5">Entfernt / Removed</td><td rowspan="5">Die betreffenden Aussagen wurden vor der Durchführung des Pretests aus dem Fragebogen entfernt, da eine zu große Anzahl an Items vorlag. Zudem waren diese Aussagen für weitere Berechnungen nicht relevant. Durch die Anpassung wurde der Fragebogen übersichtlicher gestaltet und auf die wesentlichen Aspekte fokussiert. Die Entfernung der nicht relevanten Aussagen trägt somit zu einer verbesserten Datenqualität und einer erhöhten Aussagekraft der Studie bei. / The statements in question were removed from the questionnaire before the pre-test was carried out, as there were too many items. In addition, these statements were not relevant for further calculations. The adjustment made the questionnaire clearer and focussed on the key aspects. The removal of the irrelevant statements therefore contributes to improved data quality and increased validity of the study.</td><td>---</td></tr>
<tr><td>Ich vermute, der fehlende Geruchssinn wird die Arbeit des Telenotarztes beeinträchtigen.</td><td>---</td></tr>
<tr><td>Ich vermute, die fehlende Dreidimensionalität wird die Arbeit des Telenotarztes beeinträchtigen.</td><td>---</td></tr>
<tr><td>Ich denke, dass das Konzept des Telenotarztes meine berufliche Leistung verbessert.</td><td>---</td></tr>
<tr><td>Ich denke, dass das Konzept des Telenotarztes meine Effektivität bei der Arbeit steigert.</td><td>---</td></tr>
<tr><td>Ich denke, dass der Telenotarzt auch in Situationen alarmiert wird, in denen normalerweise ein Notarzt nicht alarmiert wird.</td><td>Der Telenotarzt wird auch in Situationen alarmiert, in denen normalerweise kein Notarzt alarmiert wird./ The TEP is also alerted in situations in which an emergency physician would not normally be called.</td><td>Nachdem Teilnehmer des Pretests Verständnisprobleme bezüglich der Originalaussage angegeben hatten, wurde diese in eine verständlichere Version geändert. / After participants in the pre-test indicated comprehension problems with the original statement, it was changed to a more comprehensible version.</td><td>The TEP is also alerted in situations in which an emergency physician would not normally be called.</td></tr>
<tr><td>---</td><td>Durch das Telenotarztsystem wird die Ressource des physischen Notarztes geschont. / The TEP system conserves the resources of the physical emergency physician.</td><td>Die Aussagen wurden vor der Durchführung des Pretests in den Fragebogen aufgenommen. Diese Aussagen wurden aus der Studie von Kuntosch et al. übernommen, was zudem eine Vergleichbarkeit der Ergebnisse ermöglicht. / The statements were included in the questionnaire before the pre-</td><td>The TEP system conserves the resources of the physical emergency physician.</td></tr>
</table>

---	Ich denke, dass das Telenotarztsystem zu Kosteneinsparungen im Gesundheitswesen führt. / *I think that the TEP will lead to cost savings in the healthcare system.*	*test was carried out. These statements were taken from the Kuntosch et al. study, which also allows the results to be mroe comparable.*	I think that the TEP will lead to cost savings in the healthcare system.
---	Ich denke, dass das Telenotarztsystem zu Kostensteigerungen im Gesundheitswesen führt. / *I think that the TEP system leads to increasing costs for the healthcare system.*		I think that the TEP system leads to increasing costs for the healthcare system.
Wie häufig beabsichtigen Sie, nach der Einführung der Telenotarztanwendung, diese zu benutzen?	Wie häufig beabsichtigen Sie, die Telenotarztanwendung zukünftig bei geeigneten Einsätzen zu benutzen? / *How often do you intend to use the TEP system for suitable situations in the future?*	Die Frage wurde nach der Durchführung des Pretests aufgrund von Verbesserungsvorschlägen der Teilnehmer angepasst. / *The question was adapted after the pre-test was carried out based on suggestions for improvement from the participants.*	How often do you intend to use the TEP system for suitable situations in the future?
**Teil 3 – SUS**			
Aussagen des SUS sind im Präteritum formuliert.	Aussagen des SUS sind im Präsens formuliert. / *SUS statements are formulated in the present tense.*	Die Aussagen des SUS wurden im Präsens formuliert, da es sich um eine gegenwärtige und zukünftige Anwendung des Telenotarztsystems im MTK handelt und nicht um eine vergangene Anwendung. / *The statements of the SUS were formulated in the present tense, as it is a present and future application of the telemedicine system in the MTK and not a past application.*	SUS statements are formulated in the present tense.
**Teil 4 – Offene Fragen**			

---	Wenn Sie an Ihre letzte Nutzung des Telenotarztsystems denken: Was hat gut funktioniert? / *If you think back to the last time you used the telemedicine system, what worked well?*	Die offenen Fragen wurden vor der Durchführung des Pretests in den Fragebogen integriert. Sie wurden aus dem Interviewleitfaden der Studie von Sauers-Ford et al. übernommen und dienen der qualitativen Bewertung der Akzeptanz, Usability und Effektivität des Telenotarztsystems. / The open questions were integrated into the questionnaire before the pre-test was carried out. They were taken from the interview guidelines of the study by Sauers-Ford et al. and serve to qualitatively evaluate the acceptance, usability and effectiveness of the telemedicine system.	If you think back to the last time you used the telemedicine system, what worked well?
---	Was würden Sie bei Ihrer nächsten Nutzung anders machen? / *What would you do differently the next time you use it?*		What would you do differently the next time you use it?
---	Wenn Sie Änderungen am Telenotarztsystem oder -prozess vornehmen könnten, welche System- oder Prozessänderungen würden Sie vornehmen und warum? / *If you could make changes to the telemedicine system or process, what system or process changes would you make and why?*		If you could make changes to the telemedicine system or process, what system or process changes would you make and why?
---	Wie entscheiden Sie, ob Sie das Telenotarztsystem nutzen? Welche Diagnosen oder Probleme sind für Sie ausschlaggebend? / *How do you decide whether to use the telemedicine system? Which diagnoses or problems are decisive for you?*		How do you decide whether to use the telemedicine system? Which diagnoses or problems are decisive for you?
Was erhoffen und wünschen Sie sich von der Einführung einer Telenotarztanwendung?	Was erhoffen und wünschen Sie sich von der hessenweiten Einführung einer Telenotarztanwendung?/ What are your hopes and wishes for the introduction of a tele-emergency	Die Fragen wurden vor der Durchführung des Pretests in Absprache mit dem Referent für Rettungsdienst des HMSI geändert, aufgrund der geplanten hessenweiten Einführung einer TNA-Anwendung. Dadurch wird die Relevanz der Studie gestärkt. / The questions were changed before the pre-test was carried out in consultation with members of the	What are your hopes and wishes for the introduction of a tele-emergency doctor application throughout Hesse?

	doctor application throughout Hesse?	HMSI due to the planned introduction of a TNA application throughout Hesse. This increases the relevance of the study.	
Was befürchten Sie für negative Konsequenzen durch die Einführung einer Telenotarztanwendung?	Welche negativen Konsequenzen befürchten Sie durch die hessenweite Einführung einer Telenotarztanwendung? / *What negative consequences do you fear from the introduction of a tele-emergency doctor application throughout Hesse?*		What negative consequences do you fear from the introduction of a tele-emergency doctor application throughout Hesse?
