# Supplementary material for: Improving the introduction of telemedicine in pre-hospital emergency medicine: understanding users and how acceptability, usability and effectiveness influence this process
Source: BMC Emerg Med. 2024 Jul 12;24:114. doi: 10.1186/s12873-024-01034-6 (PMC11241972; doi:10.1186/s12873-024-01034-6)
Supplement: Supplementary file 2 — Supplementary Material 2 [file 12873_2024_1034_MOESM2_ESM.docx]

**Title Page**

**Title: Improving the introduction of telemedicine in pre-hospital emergency medicine:**

**Understanding users and how Acceptability, Usability and Effectiveness influence this process**

**Supplement 1**

Authors:

**Dr. Seán O’Sullivan***

*Researcher, Faculty of Health Sciences*

*Technische Hochschule Mittelhessen, Gießen, Germany*

*+49 641 309-6601*

*sean.o.sullivan @ges.thm.de*

Co-Authors:

**Jennifer Krautwald M.Sc.**

*Researcher, Faculty of Health Sciences*

*Technische Hochschule Mittelhessen, Gießen, Germany*

*+49 641 309-6601*

*sean.o.sullivan @ges.thm.de*

**Prof. Dr. Henning Schneider**

*Dean and Professor, Faculty of Health Sciences*

*Technische Hochschule Mittelhessen, Gießen, Germany*

*+49 641 309-6601*

*Henning.schneider@ges.thm.de*

***corresponding author**

**Methods**

Factor Analysis

To check the suitability of the variables bivariate correlation, Kaiser-Meyer-Olkin-(KMO) and Bartlett-Test were performed prior to the EFA.

Pearson's correlation coefficient was initially conducted to analyse correlations between the provided variables. The size of effect was defined according to cohen [1].

Using KMO Measure of Sampling Adequacy is a statistical method that indicates a proportion of variance in variables that might be caused by underlying factors. A high value, considered close to 1.0, generally indicates that FA may be useful with the provided data. If the value is less than 0.50, results of the FA probably won’t be very useful [2].

Bartlett’s Test of Sphericity was also performed and significance was defined at *p < .05* [3,4].

The number of factors was extracted according to the Kaiser-Kriterium, Screeplots and the interpretability of the solution [5]. For the Screeplot and kaiser criterion only factors with an eigenvalue larger than 1 were accepted. As this study was also based on the results from Sauers Ford et al. the investigators suspected 3 factors.

For the factor interpretation an orthogonal Varimax-Rotation was performed. Each variable was assigned to the factor that showed the highest loading.

Factor loading < 0,30 was not accepted. If a variable not provided a loading that was larger than 0,30 it would be rejected and a new analysis performed. Minimal acceptability was in the range of 0,30 - 0,40. Although higher loadings would be desired especially with a reduced number of available variables. To improve the reportability of a factor at least 4 variables should show a loading of 0,6 or larger. This would improve the robustness of the assigned variable to the defined factor.

If crossloading was seen, which we defined at values ranging from 0,3-0,4 for multiple factors, an assignment to a factor would be performed by using the difference between the loading of the variables. If the difference is > 0,2 the variable would be assigned to the stronger associated factor, otherwise the variable would be excluded from further analysis.

To further differentiate the analysed factors, a sub scale was then established and a reliability analysis including cronbachs alpha performed.

Each sub-scale was used for a correlation and a multiple regression analysis. The size of effect was defined according to cohen [1,6]

**Results**

Acceptability - Usability - Effectiveness

The Kaiser-Meyer-Olkin measure verified the sampling adequacy for the analysis, KMO = *.830*. Bartlett’s test of sphericity v2 (210) = *730.183, p < .001*, indicating that correlation structure is adequate for factor analyses.

Two items „The TEP is also alerted in situations in which an emergency physician would not normally be called.“ (MSA - Value: *.326*) and „The TEP assumes higher−level supervisory and control functions.“ (MSA - Value: *.350*) were excluded beforehand as the MSA Values were *< .5*.

A rotated component matrix analysis was performed and showed that loading of all variables was *> .30*. Therefore all variables could be assigned to a factor.

Two variables existed for the items ”The TEP system supports the referral of patients to the appropriate treatment centre“ with a loading of .*344* for factor 1 und a loading of .*336* for factor 3. Also the loading of the item “The TEP system reduces my workload.“ with *.372* for factor 1 and *.398* for factor 3 existed. As crossloading was seen for these variables and the difference was < *.2* these variable were excluded from further analysis.

Therefore the Kaiser-Meyer-Olkin measure was repeated and verified the sampling adequacy for the analysis, KMO = *.834*. As well as Bartlett’s test of sphericity v2 (171) = *672.248, p < .001*, indicating that correlation structure is adequate for factor analyses.

|  | Faktor | | |
| --- | --- | --- | --- |
| Variable | ***1***  *Usability* | **2**  *Effectiveness* | ***3***  *Acceptability* |
| The TEP system provides support in finding a diagnosis | *0,708* |  |  |
| The TEP system is useful for my work | *0,707* |  |  |
| The TEP system leads to an improvement in treatment options | *0,706* |  |  |
| I think that the TEP system improves the quality of patient care | *0,664* |  |  |
| I think that the TEP system increases diagnostic certainty | *0,660* |  |  |
| The TEP acts as a supportive counsellor | *0,521* |  |  |
| The TEP system leads to a delay at the scene of the emergency |  | *-0,700* |  |
| The TEP system leads to faster transport capability | *0,361* | *0,693* |  |
| The TEP system leads to significant time savings | *0,330* | *0,686* |  |
| I think that the TEP will lead to cost savings in the healthcare system |  | *0,674* |  |
| The TEP system conserves the resources of the physical emergency physician |  | *0,597* |  |
| I think that the TEP system leads to increasing costs for the healthcare system |  | *-0,567* |  |
| The TEP system leads to a faster start of treatment |  | *0,461* |  |
| The TEP system enables outpatient care for patients |  | *0,471* |  |
| The TEP system increases my workload |  |  | *-0,832* |
| The TEP system increases my documentation effort |  |  | *-0,783* |
| The TEP system disrupts the established structure of the emergency medical service |  |  | *-0,705* |
| I can imagine continuing to work with a TEP system | *0,326* | *0,308* | *0,510* |
| I think the TEP system is sensible |  |  | *0,478* |

Table 3 MSA Values and Factors

Literature

1. Cohen J. Statistical Power Analysis. Curr Dir Psychol Sci. 1. Juni 1992;1(3):98–101.

2. Kaiser HF. An index of factorial simplicity. Psychometrika. März 1974;39(1):31–6.

3. Bartlett MS. The Effect of Standardization on a χ2 Approximation in Factor Analysis. Biometrika. 1951;38(3/4):337–44.

4. Tobias S, Carlson JE. Brief Report: Bartlett’s Test of Sphericity and Chance Findings in Factor Analysis. Multivariate Behavioral Research. 1. Juli 1969;4(3):375–7.

5. Yeomans KA, Golder PA. The Guttman-Kaiser Criterion as a Predictor of the Number of Common Factors. Journal of the Royal Statistical Society Series D (The Statistician). 1982;31(3):221–9.

6. Selya AS, Rose JS, Dierker LC, Hedeker D, Mermelstein RJ. A Practical Guide to Calculating Cohen’s f2, a Measure of Local Effect Size, from PROC MIXED. Frontiers in Psychology [Internet]. 2012 [zitiert 22. Februar 2024];3. Verfügbar unter: https://www.frontiersin.org/journals/psychology/articles/10.3389/fpsyg.2012.00111
